# High Efficiency Targeting of Non-coding Sequences Using CRISPR/Cas9 System in Tilapia

**DOI:** 10.1534/g3.118.200883

**Published:** 2018-11-27

**Authors:** Minghui Li, Xingyong Liu, Shengfei Dai, Hesheng Xiao, Deshou Wang

**Affiliations:** Key Laboratory of Freshwater Fish Reproduction and Development (Ministry of Education), Key Laboratory of Aquatic Science of Chongqing, School of Life Sciences, Southwest University, Chongqing, 400715, China

**Keywords:** CRISPR/Cas9, non-coding sequence, ssDNA, germline transmission

## Abstract

The CRISPR/Cas9 has been successfully applied for disruption of protein coding sequences in a variety of organisms. The majority of the animal genome is actually non-coding sequences, which are key regulators associated with various biological processes. In this study, to understand the biological significance of these sequences, we used one or dual gRNA guided Cas9 nuclease to achieve specific deletion of non-coding sequences including microRNA and 3′ untranslated region (UTR) in tilapia, which is an important fish for studying sex determination and evolution. Co-injection of fertilized eggs with single gRNA targeting seed region of miRNA and Cas9 mRNA resulted in indel mutations. Further, co-injection of fertilized eggs with dual gRNAs and Cas9 mRNA led to the removal of the fragment between the two target loci, yielding maximum efficiency of 11%. This highest genomic deletion efficiency was further improved up to 19% using short ssDNA as a donor. The deletions can be transmitted through the germline to the next generation at average efficiency of 8.7%. Cas9-*vasa* 3′-UTR was used to increase the efficiency of germline transmission of non-coding sequence deletion up to 14.9%. In addition, the 3′-UTR of the *vasa* gene was successfully deleted by dual gRNAs. Deletion of *vasa* 3′-UTR resulted in low expression level of *vasa* mRNA in the gonad when compared with the control. To summarize, the improved CRISPR/Cas9 system provided a powerful platform that can assist to easily generate desirable non-coding sequences mutants in non-model fish tilapia to discovery their functions.

It is well-known that the protein coding sequences constitute only about 2–3% of the genome in animals and 70–90% of the animal genomes are comprised of the non-coding sequences, such as 5′-untranslated regions (5′-UTRs), intron, 3′-untranslated regions (3′-UTRs), ncRNAs such as miRNAs, lncRNAs, small interfering RNAs and PIWI-interacting RNAs (Kapranov *et al.* 2007; Amaral *et al.* 2008). Previous studies showed that large genomic deletions could be generated in zebrafish using a dual TALEN strategy (Xiao *et al.*, 2013; Gupta *et al.*, 2013; Lim *et al.*, 2013; Liu *et al.*, 2013). Recently, CRISPR/Cas9 has been extensively used to generate small insertion and deletion (indel) mutations to disrupt the open reading frames (ORF) of the protein coding genes (Friedland *et al.* 2013; Ren *et al.* 2013; Chang *et al.* 2013; Cong *et al.* 2013; Li *et al.* 2014). However, it is challenging to use CRISPR/Cas9 for the mutation of non-coding sequences because small indels caused by a single mutation are impossible to yield loss of function or some limitations of target selection. It has been reported that large genomic deletion could be achieved using dual guide RNAs (gRNAs) system in mammalian cells and animal models such as mouse and zebrafish (Wang *et al.* 2015; Zhu *et al.* 2016; Zhao *et al.* 2017; Xiong *et al.* 2018). There is no report showing that non-coding sequences deletion could be produced in non-model fish by CRISPR/Cas9 system.

Recently, single strand DNA (ssDNA) oligonucleotide has been used as donor template in combination with the engineered nucleases for efficient genome editing (Radecke *et al.* 2010; Chen *et al.* 2011). ssDNA mediated knocking in mammalian cells occurs via homology-directed repair (HDR) and is more efficient than using double-stranded donor plasmids. Target genomic deletion of up to 100 kb has been achieved in mammalian cells using ssDNA oligonucleotides in tandem with zinc finger nucleases (ZFNs) (Chen *et al.* 2011). Recent investigations showed that large deletions can be induced by incorporating single-strand oligodeoxynucleotides together with dual gRNAs in *C. elegans* (Paix *et al.* 2014; Arribere *et al.* 2014). In these studies, the efficiency of large DNA fragment deletion was significantly increased by using ssDNA mediated end joining when compared to that without ssDNA. Up to now, deletion of miRNA via CRISPR/Cas9 system has only been reported in zebrafish model (Xiao *et al.* 2013; Narayanan *et al.* 2016; Xiong *et al.* 2018). However, it is not known whether the efficiency of large DNA fragment deletion could be improved using ssDNA and CRISPR/Cas9 system in fish?

Nile tilapia (*Oreochromis niloticus*), a gonochoristic teleost with an XX/XY sex determination system, is regarded as one of the most important species in global aquaculture (FAO, 2013). In addition, it is also considered as an important laboratory model for understanding the developmental genetic basis of sex determination and evolution (Brawand *et al.* 2014; Beardmore *et al.* 2001). Earlier, we reported that successful application of CRISPR/Cas9 technology in tilapia gene disruption (Li *et al.* 2014, Li and Wang 2017). It was observed that homozygous mutation of *amhy*, *amhrII*, or *gsdf* in XY fish resulted in male to female sex reversal (Li *et al.* 2015; Jiang *et al.* 2016). In contrast, homozygous mutation of *foxl2* or *cyp19a1a* in XX fish resulted in female to male sex reversal (Zhang *et al.* 2017). In recent years, it has been reported that non-coding sequences play an important role in controlling many physiology process including sex determination. For example, in silkworm *Bombyx mori*, a single female-specific piRNA was found to be responsible for primary sex determination (Kiuchi *et al.* 2014). In mouse, deletion of Enh13, a 557–base pair element located 565 kb 5′ of *Sox9*, resulted in XY females mouse with *Sox9* transcript levels equivalent to XX gonads (Gonen *et al.* 2018). It is noteworthy that promoter variations contributed to sex determination role of some genes in fish, such as *gsdfy* in *Oryzias luzonensis* and *sox3y* in *Oryzias dancena* (Myosho *et al.* 2012; Takehana *et al.* 2014). Therefore, the objective of this study was to explore the potential of the CRIPSR/Cas9 technology for engineering large genomic deletions of non-coding sequences, such as miRNA and 3′-UTR, in a farmed fish tilapia. This technique will facilitate studies on the roles of non-coding sequences in tilapia sex determination.

## Materials And Methods

### Fish maintenance

Nile tilapias (*Oreochromis niloticus*) were preserved in re-circulating freshwater tanks at 26° before using them for the experiments. All-XX progenies were obtained by crossing a pseudo male (XX male, producing sperm after hormonal sex reversal) with a normal female (XX). All-XY progenies were obtained by crossing a super male (YY) with a normal female (XX). Animal experiments were conducted in accordance with the regulations of the Guide for Care and Use of Laboratory Animals and were approved by the Committee of Laboratory Animal Experimentation at Southwest University (Chongqing, China).

### gRNA design and in vitro transcription

CRISPR/Cas9 targets were selected using ZiFiT Targeter software online (http://zifit.partners.org/ZiFiT). The gRNA target sites were designed using sequences on the sense or antisense strand of DNA (Chang *et al.* 2013). For miRNA125 disruption, the target site was selected in the seed sequences. Further, for deletion of large DNA fragment, target sites were selected in the up and downstream of the seed sequences. Basic local alignment search tool (BLAST) was performed with the tilapia genome to avoid off-targets. In addition, a restriction enzyme cutting site adjacent to the NGG PAM sequence was selected for convenient mutagenesis analysis through digestion of the fragment amplified with primers covering the target.

For gRNA *in vitro* transcription, the DNA templates were obtained from pMD19-T gRNA scaffold vector (kindly provided by Dr. JW Xiong, Peking University, China) using polymerase chain reaction (PCR) amplification (Chang *et al.* 2013). Forward primer consisted of T7 polymerase binding site, 20 bp gRNA target sequence and 5′ partial sequence of gRNA scaffold. Reverse primer was designed based on the 3′ sequence of gRNA scaffold. The reaction mixture (25μl) consisted of 2 μl of gRNA scaffold template, 2.5 μl of 10×reaction buffer (MgCl_2_^+^), 0.8 mM of each dNTPs, 0.2 μM of each primer, and 0.25 unit of TaKaRa Ex Taq DNA polymerase (Takara, Japan). The PCR conditions were 94° for 3 min; 35 cycles of 15 s at 94°, 30 s at 60°, and 1 min at 72°); and a final extension of 10 min at 72°. The PCR products were purified using QIAquick Gel Extraction Kit (Qiagen, Germany) following the manufacturer′s instructions. *In vitro* transcription was performed with Megascript T7 Kit (Ambion, USA) for 4 hr at 37° using 300 ng of purified DNA as template. The transcribed gRNA was purified through phenol/chloroform extraction and isopropanol precipitation, and quantified using NanoDrop-2000 (Thermo Scientific, USA) before diluting to 300 ng/μl in RNase free water and then stored at -80° until further use.

### Cas9 mRNA in vitro transcription

The Cas9 nuclease expression vector pcDNA3.1+ (Invitrogen, USA) used for *in vitro* transcription of the Cas9 mRNA has been described previously (Chang *et al.* 2013). Plasmids were prepared using a plasmid midi kit (Qiagen, Germany), and linearized with *Xba* I and purified by ethanol precipitation as corresponding transcription templates. Cas9 mRNA was generated via *in vitro* transcription of 1 μg linearized template DNA with a T7 mMESSAGE mMACHINE Kit (Ambion, USA) following the manufacturer′s instructions. The resultant mRNA was purified using MegaClear Kit (Ambion, USA) before resuspension in RNase-free water (Qiagen, Germany) and quantified using NanoDrop-2000 (Thermo Scientific, USA).

### Construction of Cas9-vasa 3′-UTR expression vector

The Nile tilapia *vasa* 3′-UTR (280 bp) was amplified by PCR using its cDNA clone as template with forward primer (5′- GCGGCCGCGAGCAGCGCAGTCACACAGCAATG-3′, underline represents the *Not* I) and reverse primer flanking the poly A tail (5′-GTCGACGGCCGAGGCGGCCGACATG-3′, underlined nucleotides represents *Sal* I). The reaction was performed using TaKaRa Ex Taq DNA polymerase (Takara, Japan) following the manufacturer’s instructions. The *vasa* 3′-UTR was inserted into the downstream of Cas9 ORF through restriction enzyme digestion and ligation with T4 ligase. Chimeric RNA was synthesized via *in vitro* transcription as described above.

### Microinjection and genomic DNA extraction

Combinations of single or two gRNA with Cas9 mRNA were microinjected into tilapia embryos at one-cell stage. To improve the large genomic deletion efficiency, ssDNA with different lengths of left and right homology arms located at the outer sides of the Cas9 cutting edges were used that were synthesized by Invitrogen Company (Shanghai, China). gRNA (150 ng/μl) and Cas9 mRNA (500 ng/μl) or Cas9-*vasa* 3′-UTR mRNA (500 ng/μl) were mixed and injected to the embryos in combination with and without ssDNA (20 ng/μl). The injected embryos were hatched at 26° and collected at 72 hr after injection and incubated for 1 hr at 55° in 100 μl lysis buffer containing 0.5% sodium dodecyl sulfate (SDS), 25 mM ethylenediaminetetraacetic acid (EDTA) (pH 8.0), 10 mM Tris-HCl (pH 8.0), and 20 mg/ml of proteinase K. The lysate was extracted with phenol/chloroform and precipitated with ethanol. The extracted DNA was quantified using NanoDrop-2000 and used as a template.

### Mutation assay

Genomic DNA fragments covering the target site were amplified using gene specific primers to detect miRNA125 seed sequence mutation. The PCR products were purified using QIAquick Gel Extraction Kit (Qiagen, Germany). After restriction enzyme digestion (RED) with *Mse* I, the uncleaved DNA bands were obtained and cloned into pGEM-T easy vector (Promega, USA) for sequencing. The percentage of uncleaved DNA band (potential mutations in target site) was calculated by measuring the band intensity (uncleaved DNA band intensity/total DNA band intensity) using Quantity One Software (Bio-Rad, USA) (Henriques *et al.* 2012).

For some targets devoid of suitable restriction enzyme, T7 endonuclease I (T7EI) assay was performed following previously described protocol (Reyon *et al.* 2012). Briefly, the genomic DNA was amplified using PCR with primers under following conditions: 95° for 5 min, 95–85° at −2°/s, 85–25° at −0.1°/s, hold at 4° (The primers are listed in Table S1). The PCR products were digested with T7E1 (NEB, China) and analyzed on an ethidium bromide-stained TAE gel.

Genomic DNA fragments covering the two target sites were amplified using gene specific primers to detect large fragment deletion. Two pairs of primers (miR200-F1/miR200-R1; and miR429-F1/miR429-R1) were designed for detection of miRNA200a/200b and miRNA429a deletions respectively. miR200-F1 and miR429-R1 were used for detection of miRNA200a/200b/429 deletion. One pair of primers (*vasa*-3′-UTR-S-F1/*vasa*-3′-UTR-S-R1) was designed to detect *vasa*-3′-UTR deletion. All these primers used in these studies are listed in Table S1. The PCR products including wild type and mutated DNA bands were purified using QIAquick Gel Extraction Kit (Qiagen, Germany) and cloned into pGEM-T easy vector and transformed into DH5α competent cells. Fifty colonies were randomly selected and screened by PCR. The positive clones were sequenced and then aligned with the wild type sequences to determine whether the interval fragments were deleted. The deletion frequency was determined by calculating number of mutated clones *vs.* total clones sequenced.

For calculating deletion efficiency of each fish, F0 fish injected with normal Cas9 mRNA were first screened by PCR amplification of genomic DNA extracted from a piece of caudal fin. Among the mutants, eight fish, which have expected DNA bands with stronger intensity compared with other fish, was selected to calculate the mutation frequency in each fish as described above. For detection of F0 fish injected with Cas9-*vasa* 3′-UTR mRNA, genomic DNA was extracted from gonad tissue at 90 days after hatching (dah). PCR amplification was performed to examine the deletions induced by gRNA/Cas9-*vasa* 3′-UTR as described above.

### Detection of heritable mutations

Fifty fishes injected with gRNA targeting miRNA200a/200b/429a, Cas9 mRNA and ssDNA were screened by PCR amplification of genomic DNA extracted from semen collected from each fish at 240 dah and designated as founders to investigate whether CRISPR/Cas9-mediated deletion of non-coding sequences can transmit to subsequent generations. The positive F0 XY fish was upraised to sexual maturity and followed by mating with wild type tilapia. F1 larvae were collected at 10 dah and genotyped by PCR amplification using genomic DNA extracted from each F1 larva. The positive individual clones were further confirmed by DNA sequencing.

For examination of the germline transmission in fishes injected with gRNA targeting miRNA200a/200b/429a, Cas9-*vasa* 3′-UTR mRNA and ssDNA, forty F0 XY fishes were randomly selected and upraised to sexual maturity. The semen of each XY fish was collected artificially at 240 dah. PCR amplification with genomic DNA extracted from these sperm was performed to examine whether deletions induced in the sperm. F1 offsprings were obtained by F0 positive founders mated with wild type XX female fish. For each founder, F1 larvae were collected at 10 dah and genotyped by PCR amplification using genomic DNA extracted from each F1 larva. Among the F0 fish, four fish with the highest germline transmission efficiency was selected as the efficiency of germline transmission using Cas9-*vasa* 3′-UTR construct.

### Real-time PCR

Three parallel samples (each composed of gonads from 6 individuals) were obtained from *va*sa 3′-UTR mutant XY fish and control XY fish at 90 dah. Total RNAs were extracted and treated with DNase I to eliminate genomic DNA contamination. First strand cDNAs were synthesized using PrimeScript RT Master Mix Perfect Real Time Kit following the manufacturer’s instructions (Takara, Japan). RT-qPCR was performed on an ABI-7500 real-time PCR machine according to the protocol of SYBR Premix Ex TaqTM II (Takara, Japan). Primer sequences used for RT-qPCR are listed in Table S1. The relative abundances of mRNA transcripts were evaluated using the formula: R = 2^-△△Ct^ (Livak and Schmittgen 2001). *β-actin* and *nanos1* was used as the internal control.

### Statistics

Data are expressed as the mean ± SD. Differences in the data between groups were tested by independent-samples *t*-test in the SPSS software. *P* < 0.05 was considered to be significantly different.

### Data availability

Fish strains and plasmids are available upon request. The authors affirm that all data necessary for confirming the conclusions of the article are present within the article, figures, tables, and supplemental information. Supplemental material available at Figshare: https://doi.org/10.25387/g3.7331054.

## Results

### Disruption of miRNA125 by targeting the seed sequence using single gRNA

**Figure 1:**
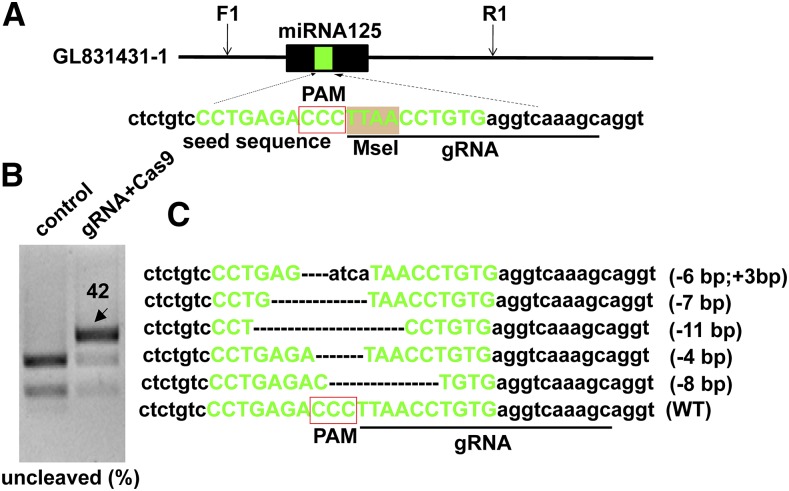
Efficient disruption of miRNA125 by targeting the seed sequence using single gRNA. (A) The miRNA125 were selected as target to demonstrate the feasibility of CRISPR/Cas9 mediated mutagenesis. gRNA was designed in the seed sequence (green letters) containing a restriction enzyme cutting site (gray). The indels (insertion and deletion) were confirmed with restriction enzyme digestion and Sanger sequencing. (B) Two cleavage bands were detected in control group, while an intact DNA fragment (indicated by arrow) was observed in embryos injected with both Cas9 mRNA and target gRNA. The percentage of uncleaved band was measured by Quantity One Software. (C) Representative Sanger sequencing results from the uncleaved bands are listed. Deletions and insertions are marked by dashes and lowercase letters, and the protospacer adjacent motif (PAM) is highlighted in box. Numbers to the right of the sequences indicate the loss or gain of bases for each allele, with the number of bases inserted (+) or deleted (−) indicated in parentheses. WT, wild type.

Several studies have demonstrated that the miRNA seed region is critical for miRNA-mRNA pairing and indels in this region would interfere with its function (Bartel 2009). Thus, the CRISPR target was designed in the seed sequence of mature miRNA. Tilapia miRNA125 was selected as the target to examine whether mutation could be induced in the seed region using CRISPR/Cas9. gRNA containing restriction enzyme *Mse* I was designed in the seed sequence of miRNA125 (Figure 1A). Co-injection of gRNA and Cas9 mRNA led to indels formation in the seed region. The indels were confirmed with restriction enzyme digestion and Sanger sequencing. *Mse* I-mediated digestion generated two small DNA fragments in the control group, while an intact DNA fragment was detected in embryos injected with gRNA and Cas9 mRNA, indicating indels were induced in the target site. The mutation frequency was found to be approximately 42% in the pools of 20 embryos (Figure 1B). The uncleaved DNA band was recovered for subcloning. Twenty positive clones were randomly sequenced and 17 of 20 clones were found to have indels in the seed sequence, while 3 of 20 clones were found to be wild type sequence. This showed that these mutations using single gRNA could alter the seed sequence, thus leading to loss of function of the miRNA125 (Figure 1C). Further, the restriction enzyme digestion assay showed that 12 of the 25 F0 fish (12/25, 72%) have mutations, as indicated by presence of an uncleaved DNA band. Among the 12 mutants, eight fishes, which have uncleaved DNA bands with stronger intensity compared with other fish, were selected to determine the mutation rate in each fish. A wide variation was observed in the mutation rate of F0 fishes ranging from 18 to 74% (Table S2).

### Deletion of miRNA200a/200b and miRNA429a using one pair of gRNAs

**Figure 2:**
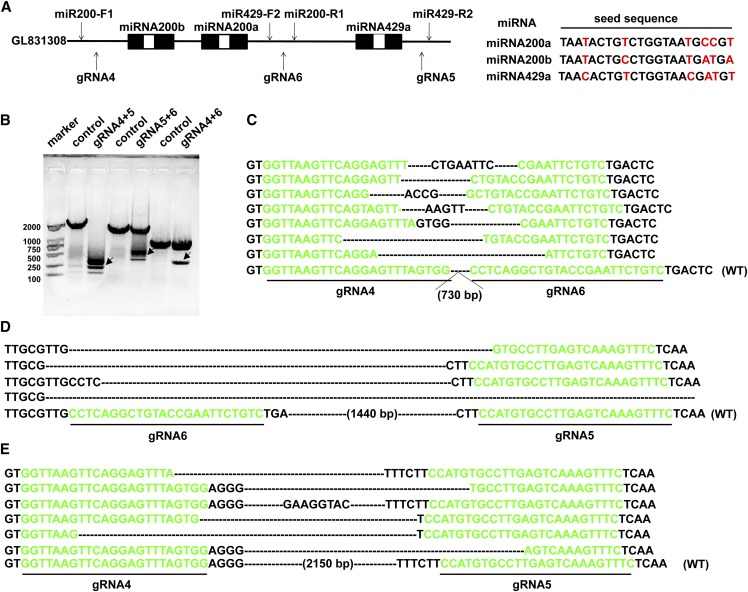
Deletion of miRNA200a/200b/429a using one pairs of gRNAs and Cas9. (A) Tilapia miRNA200b, miRNA200a and miRNA429a cluster are locate on the same chromosome. They exhibit high similarity in their seed sequences. gRNA4/6 targeting miRNA200a/b (729 bp) or gRNA5/6 targeting miRNA429a (1.4 kb), or gRNA4/5 targeting the miRNA cluster (200a/200b/429a) (2.4 kb) were designed in the up and downstream of the seed sequences. (B) PCR amplification indicated successful deletion of the fragments with expected size (indicated by arrow). (C, D, E) Sequencing of the PCR products further confirmed the deletions. Deletions are marked by dashes. WT, wild type. miR200-F1/miR200-R1 and miR429-F1/miR429-R1 were designed for detection of miRNA200a/200b and miRNA429 deletions respectively. miR200-F1 and miR429-R1 were used for detection of miRNA200a/200b/429 fragment deletion.

Tilapia miRNA200b, miRNA200a and miRNA429a cluster are located on the same chromosome exhibiting high similarity in their seed sequences (Figure 2A). Three gRNAs (gRNA1/2/3) were designed to target the seed sequences of miRNA200b, miRNA200a and miRNA429a. However, the T7E1 and Sanger sequencing did not detect any indels in the seed regions (Figure S1). Dual gRNAs (gRNA4/6 targeting miRNA200a/200b (729 bp), gRNA5/6 targeting miRNA429a (1.4 kb), and gRNA4/5 targeting the miRNA 200a/200b/429a cluster) (2.4 kb), which located at up and downstream of the seed sequence, were designed to delete the complete DNA fragment containing miRNA200a/200b/429a. PCR amplification using primers spanning the two cutting sites indicated successful deletion of the fragments with expected size (300bp for miRNA200a/200b deletion, 400bp for miRNA429a deletion, 600bp for miRNA200a/b/429a deletion) (Figure 2B). Sequencing of these PCR bands further confirmed the precise deletions detected between the two targeted sites (Figure 2C-2E). This demonstrated that the deletion of large genomic fragment was successfully achieved using CRISPR/Cas9 in tilapia.

With reference to the individual screening, PCR results indicated that 12 of the 20 miRNA200a/200b targeting fish (12/20, 60%), 10 of the 40 miRNA429a targeting fish (10/40, 25%), and 12 of the 100 miRNA200a/200b/429a targeting fish (12/100, 12%) harbored the desired large fragment deletion. Among these mutants, eight fishes were selected to calculate the deletion rate in each fish. The maximum deletion efficiency in individual fish was found to be 11% in miRNA200a/200b mutants, 9% in miRNA200a/200b/429a mutants and 12% in miRNA429a mutants. The average deletion efficiency reached is 8.3% in miRNA200a/200b mutants, 8.4% in miRNA429a mutants and 7.2% in miRNA200a/200b/429a mutants (Table 1). It was observed that the deletion efficiency significantly decreased with the increase of genomic DNA length.

**Table 1:** Fragment deletion of miRNA200a/200b/429a in each mutant induced by CRISPR/Cas9

Target	Number of F0 tested	Mutant	Frequency (%)	Fragment deletion frequency (%)								Average deletion frequency (%)
				#1	#2	#3	#4	#5	#6	#7	#8	
**miRNA200a/200b**	20	12	60	5	10	9	11	8	**11**	6	7	8.3
**miRNA429a**	40	10	25	8	7	9	10	**12**	9	6	7	8.4
**miRNA200a/200b/429a**	100	12	12	9	6	8	7	**9**	9	4	6	7.2
**miRNA200a/200b/429a+80 bp ssDNA**	50	11	22	**19**	11	8	9	12	16	5	11	11.4*

Asterisk indicates significant difference between miRNA200a/200b/429a mutants and miRNA200a/200b/429a+80 bp ssDNA mutants (*P* < 0.05).

### Increased targeting efficiency using one pair of gRNAs and ssDNA

**Figure 3:**
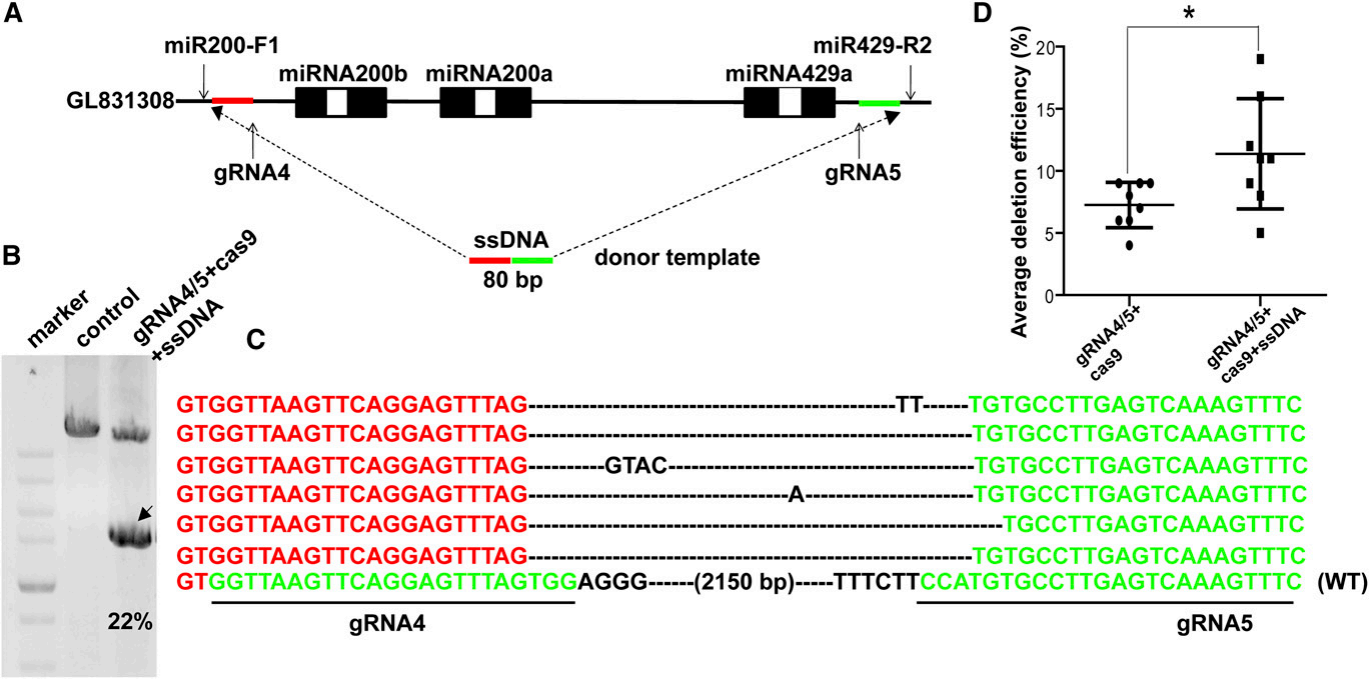
Targeting efficiency was significantly increased using one pairs of gRNAs and ssDNA donor. (A) ssDNA oligonucleotide with 80 bp left and right homology arms were co-injected with two gRNAs and Cas9 mRNA into one-cell stage tilapia embryos. (B) PCR amplification showed successful deletion of the miRNA200a/b/429a fragments (indicated by arrow). miR200-F1 and miR429-R1 were used for detection of miRNA200a/200b/429a fragment deletion. (C) Sequencing of the PCR products further confirmed the deletions. gRNA sequences are underlined. Deletions are marked by dashes. WT, wild type. (D) The average deletion efficiency of eight F0 fishes (11.4%) was significantly higher than that without using ssDNA (7.2%). Data are expressed as the mean ± SD. Differences between groups were tested by independent-samples *t*-test. Asterisk indicates significant difference between groups (*P* < 0.05).

ssDNA homologous template was used in order to further improve the DNA fragment deletion together with gRNA4/5 targeting the miRNA 200a/200b/429a cluster and Cas9 mRNA (Figure 3A). PCR amplification of genomic DNA using primers miR200-F1 and miR429-R1 showed successful deletion of the whole miRNA200a/200b/429a fragments (Figure 3B). In addition, the sequencing of the band with correct size confirmed the presence of the desired deletions in the mutant fish (Figure 3C). Individual screening showed that 11 of the 50 fish (11/50, 22%) harbored the desired large fragment deletion. The highest deletion efficiency in individual fish using ssDNA was found to be improved (∼19%) than without using ssDNA (∼9%). The average deletion efficiency of eight F0 fishes was 11.4%, which was significantly higher than that with no ssDNA (7.2%) (*P* = 0.0291, <0.05) (Figure 3D, Table 1). The length of total homology of ssDNA was reduced from 120 bases to 40 bases to determine the minimal homology requirements in the ssDNA. A significant reduction was observed using 40 bp of homology compared with other groups (Table 2). These results demonstrated that combination of ssDNA and CRISPR/Cas9 system could improve the efficiency of large genomic DNA deletion.

**Table 2:** Comparison of targeting efficiency with and without ssDNA

Target	Number of fish tested	Mutants	Frequency (%)	Highest fragment deletion rate (%) in the mutants
**gRNA4+gRNA5+Cas9**	50	6	12	9
**gRNA4+gRNA5+Cas9+ssDNA 40 bp**	50	5	10	10
**gRNA4+gRNA5+Cas9+ssDNA 60 bp**	50	8	16	12
**gRNA4+gRNA5+Cas9+ssDNA 80 bp**	50	10	20	19
**gRNA4+gRNA5+Cas9+ssDNA 120 bp**	50	11	22	18

### Deletion of 3′untranslated regions (UTRs) using one pair of gRNAs

**Figure 4:**
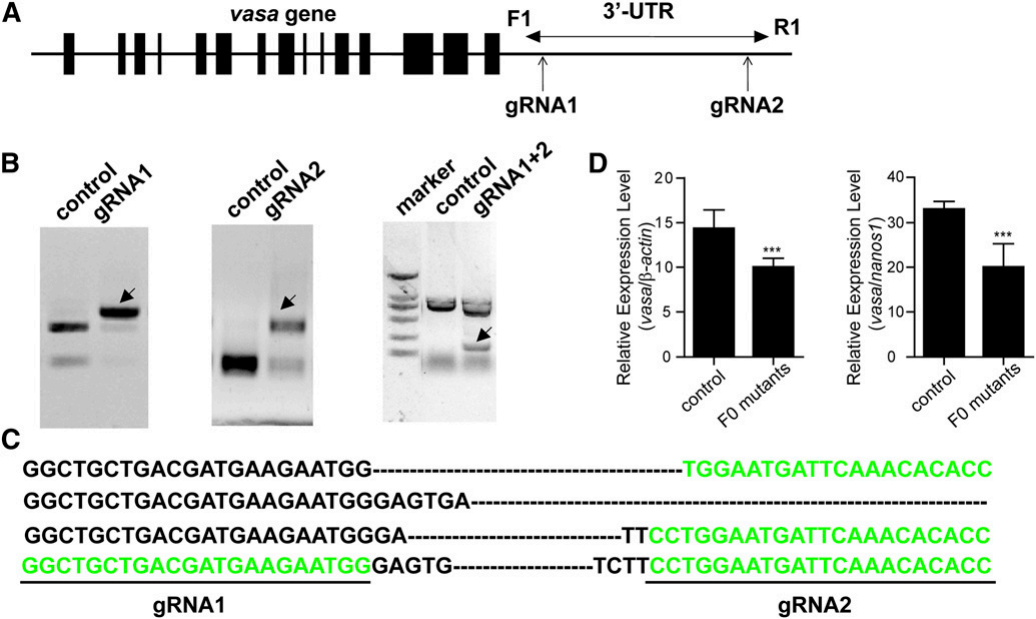
Deletion of 3′-untranslated region (UTR) using one pairs of gRNAs and ssDNA. (A) Two gRNAs was designed in the 3′-UTR of *vasa*. (B) PCR amplification of genomic DNA extracted from the injected and control embryos indicated successful deletion of the 3′-UTR region. (C) Sequencing of the PCR products further confirmed the deletions (indicated by arrow). Deletions are marked by dashes WT, wild type. (D) The *vasa* mRNA expression level obtained by real-time PCR was significantly decreased in the F0 mutants when compared with the control group. *β-actin* and germ cell marker *nanos1* were used as the internal control. The relative abundance of *vasa* mRNA transcripts was evaluated using the formula: R= 2^-△△Ct^. Data are presented as the means± SD of the triplicates. ***P < 0.01 compared with the control using one-way ANOVA.

Various studies have demonstrated that 3′-UTRs are not only the targets of miRNAs, but also could regulate the gene expression by controlling mRNA stability and translation (Anderson, 2008; Sayed and Abdellatif, 2011). PCR amplification of genomic DNA extracted from the pooled embryos (after co-injected with two gRNAs and Cas9 mRNA) indicated successful deletion of the 3′-UTR region. This deletion was further confirmed by the sequencing of the PCR products (Figure 4A-C). Moreover, individual screening showed that 7 of the 22 fishes (7/22, 31.8%) harbored the desired large fragment deletion with the overall deletion frequency was found to be 9–43% (Table S3). Real-time PCR revealed that *vasa* mRNA level was significantly reduced in 3′-UTR deletion tilapia gonads, when compared with the control group (Figure 4D).

### Germline transmission of large fragment deletions

Ten male F0 founders of miRNA200a/200b/429a mutants were screened by PCR amplification of the genomic DNA extracted from semen of each fish. Among the 11 F0 mutants produced by gRNA, Cas9 and ssDNA injection, four male founders with highest deletion frequency in the semen were selected to examine germline transmission. The results showed that all the F0 mutants transmitted the mutations to their offsprings, demonstrating that the large fragment deletions were inheritable. The ratio of F1 fish containing the fragment deletion from the four founders ranged from 5.5 to 11.9%, with an average efficiency of 8.7% (Table 3).

**Table 3:** The germline transmission of the eight founder fishes for the miRNA200a/200b/429a locus

Group	Founder fish	F1 individuals evaluated	Positive individuals	% of germline transmission
1 (Cas9)	#1	52	5	9.6
	#2	38	3	7.9
	#3	42	5	11.9
	#4	36	2	5.5
	**Average efficiency**			8.7
2 (Cas9-*vasa* 3′-UTR)	#5	42	5	11.9
	#6	33	6	18.1
	#7	38	5	13.1
	#8	24	4	16.6
	**Average efficiency**			14.9*

Asterisk indicates significant difference between group 1 and 2 mutants (*P* < 0.05).

**Figure 5:**
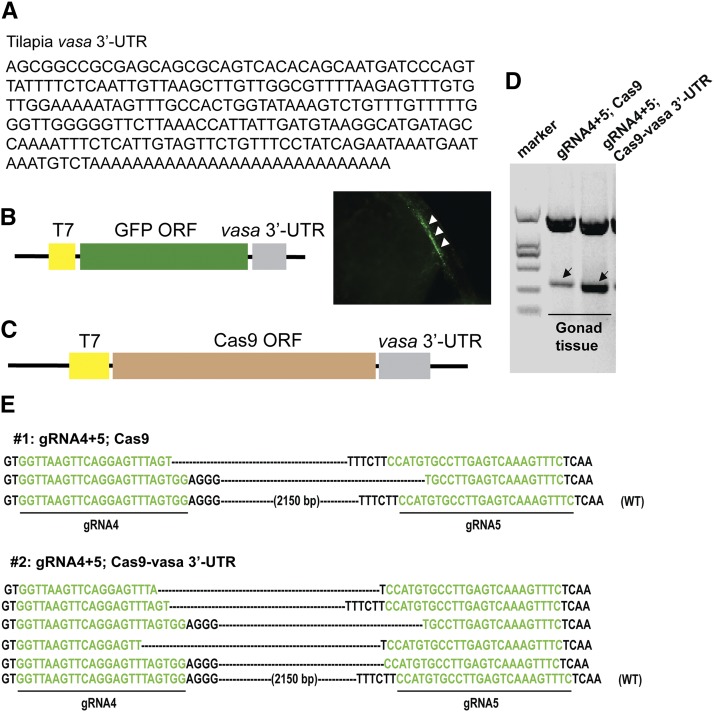
The DNA fragment deletion was enhanced using Cas9-*vasa* 3′-UTR in gonads. (A) the *vasa* 3′-UTR sequence of tilapia used in this study was listed. (B) *vasa* 3′-UTR direct GFP mRNA expression in the germ cells. GFP expressing cells indicated the germ cells (indicated by arrowhead). (C) *vasa* 3′-UTR was inserted into downstream of Cas9 open reading frame. (D) PCR amplification showed successful deletion of the target DNA in the gonad tissue (indicated by arrow). (E) Sequencing of the PCR products further confirmed the deletions. Deletions are marked by dashes. WT, wild type.

Cas9-*vasa* 3′-UTR expression vector was constructed to further improve the germline transmission of large genomic deletion. The tilapia *vasa* 3′-UTR sequence is shown in Figure 5A. *vasa* 3′-UTR of tilapia can locate the GFP protein in specifically the germ cell (Figure 5B). The *vasa* 3′-UTR was inserted into the downstream of Cas9 ORF to induce the Cas9 protein expression in the germ cells (Figure 5C). After 90 dah, PCR amplification showed successful deletion of the target fragments in pooled testis tissue of normal Cas9 (control) and Cas9-*vasa* 3′-UTR injected fish. However, the deletion efficiency in fish injected with Cas9-*vasa* 3′-UTR was found to be much higher when compared to the control fish injected with normal Cas9 (Figure 5D). These deletions were further confirmed by sequencing of the PCR products (Figure 5E). Among the 6 F0 fishes, it was observed that all the four F0 fishes selected with highest deletion efficiency transmitted the mutations to their offsprings. The ratio of F1 fish carrying the fragment deletion from the four founders (fish #5∼#8) ranged from 11.9 to 18.1%, with an average efficiency of 14.9%, which is significant higher than that using normal Cas9 mRNA (8.7%) (*P* = 0.0205, <0.05) (Table 3).

## Discussion

Here, we report a simple and efficient method to generate non-coding sequence knockout mutants using the CRISPR/Cas9 system in tilapia. First, we could successfully design highly efficient targets for miRNA125 disruption by mutation of its seed sequences. This can be attributed to the presence of indel in the seed region that can disrupt miRNA activity because seed sequence is important for target recognition and binding (Bartel 2009; Kim *et al.* 2013). It has also been reported mutation of seed region disrupt the function of miRNA in mammalian cells (Kim *et al.* 2013). Second, we showed that the co-injection of two gRNAs into tilapia embryos can induce deletion of large fragments up to 2.4 kb containing miRNA200a/200b/429a between the two targeted loci. In addition, efficiency deletion of *vasa* 3′-UTR was also achieved by using CRISPR/Cas9. In principle, gRNA target could be easily designed for any genomic DNA, in some cases, the seed region was not suitable for selection of efficient targets due to the high similarity in the seed region of miRNA family, such as miRNA200a/200b/429a described in this study. This method using two gRNA strategy described here is also useful for whole gene open reading frame or exon deletion in fish, in which functional gRNAs cannot be designed due to exon sequence constraints. To our knowledge, this is the first report showing successful deletion of non-coding sequence in non-model fish.

Co-injected two gRNAs, Cas9 mRNA and ssDNA resulted into improved deletion efficiency in tilapia. This is in consistent with the study where co-transfected ssDNA was found to support the ligation of DSBs induced by ZFNs in mammalian cells (Chen *et al.* 2011). In the present study, the average deletion frequency examined in the caudal fin tissue was 11.4%, which is significant higher that that without using ssDNA (7.2%). The efficiency was obtained by counting number of mutated clones *vs.* total clones sequenced. Mutant sequences are preferred over the wild type sequence during PCR amplification because of the differences in length. Additionally, short DNA fragment (mutated DNA fragment) was easily ligated to the vector when compared with the wild type fragment. Therefore, the deletion efficiency calculated by this method was higher than the actual deletion efficiency. This is a non-quantitative method for detecting deletions. Nonetheless, this is the first report demonstrating improved efficiency of large genomic fragment deletion in fish species.

Germline transmission is critical to obtain the homozygous knockout animals. Although the frequency of large fragment deletion with two gRNAs is much lower than that of the single site indel mutation induced by CRISPR/Cas9, these large deletions could also be transmitted through the germline. It has been reported that the efficiency of large genomic deletions in germ cells was found to be significantly increased using TALEN-*nanos*-3′UTR construct because *nanos*-3′UTR can direct Cas9 protein in the germ cells specifically (Liu *et al.* 2013). Our studies demonstrated that 3′-UTR of *vasa* gene can localize the target mRNA to primordial germ cells specifically during tilapia embryo development (Li *et al.* 2014). To further improve germline transmission of large genomic deletion, Cas9-*vasa* 3′-UTR vector was first constructed and used in this study to increase the efficiency of germline transmission of non-coding sequence deletion. It was observed that four F0 founders transmitted germline mutations to an average of 14.9% of their progeny, which is a great improvement in efficiency when compared to the methods based on normal Cas9 expression (8.7%). Therefore, the germline transmission could be significantly improved using our constructed Cas9-*vasa* 3′-UTR vector, which is mainly caused by directing the Cas9 protein in germ cells during embryo development. This strategy was also applicable to increase germline transmission of gene open reading frame mutations.

To the best of our knowledge, this study represents the first description of successful homology direct repair in tilapia using ssDNA as a donor template in *vivo*. Recently, efficient targeted gene knock-in by combining CRISPR/Cas9 with ssDNAs was achieved in rats and mice (Wefers *et al.* 2013; Yang *et al.* 2013; Yoshimi *et al.* 2016). TALEN-mediated genetic elements insertion using ssDNA oligonucleotides has also been reported in zebrafish (Bedell *et al.* 2012). In the future, targeted exogenous gene insertion in tilapia for transgenesis would be achieved by using CRISPR/Cas9 with ssDNA. The use of ssDNA can also facilitate an array of genome changes, including the introduction of single-nucleotide polymorphisms (SNP), *loxP*, and other defined small genetic elements for basic research applications in tilapia.

In conclusion, this study established an efficient approach for the generation of precise large genomic deletions using CRISPR/Cas9 system in non-model fish tilapia. Further, it was demonstrated that the use of two gRNAs in combination with ssDNA could increase the deletion efficiency. The germline transmission was also improved by directing Cas9 protein in germ cells via *vasa*-3′-UTR. This study provides a robust and cost-effective tool to study the role of non-coding sequences *in vivo* in tilapia. The method described in this study may also be applicable to other non-model fish species. This platform will also help identify new genetic elements involved in tilapia sex determination.
